# DNA Photofluids: An Innovative Breakthrough in Mimicking Cellular Life Behaviors

**DOI:** 10.34133/research.0845

**Published:** 2025-08-21

**Authors:** Rui Wang, Wenguo Cui, Xinliang Chen

**Affiliations:** ^1^ The International Peace Maternity and Child Health Hospital, School of Medicine, Shanghai Jiao Tong University, Shanghai 200030, China.; ^2^ Shanghai Key Laboratory of Embryo Original Diseases, Shanghai 200030, China.; ^3^ Department of Orthopaedics, Shanghai Key Laboratory for Prevention and Treatment of Bone and Joint Diseases, Shanghai Institute of Traumatology and Orthopaedics, Ruijin Hospital, Shanghai Jiao Tong University School of Medicine, Shanghai 200025, China.

## Abstract

Current molecular machines face substantial challenges in coordinating their actions in space and time to generate cell-like macroscopic motions. A recent study in *Nature Materials* introduced a light-responsive artificial DNA nanomachine based on liquid–liquid phase separation technology—photofluids. By applying different light stimuli for spatiotemporal control, this nanomachine system successfully mimics typical cellular behaviors such as division, deformation, pseudopod extension, and rotation at the macroscopic scale for the first time. This study represents an innovative pathway from energy conversion at the molecular level to cell-like motion at the macroscopic level.

Molecular machines are structures operating at the molecular scale that can temporarily deviate from equilibrium under external stimuli, generating mechanical motion-like responses [1]. In nature, cells rely on various biomolecular machines to convert light or chemical energy into complex and precise mechanical movements, achieving dynamic non-equilibrium behaviors such as shape transformation, migration, and division [2]. Researchers have long sought to design and synthesize artificial molecular machines that can replicate such natural cellular behaviors [3,4]. Existing artificial molecular machines can already simulate simple biological functions, such as the ribosome picking up amino acids [5], and muscle contraction [6]. However, these approaches remain immature. Most operate in isotropic, viscously dissipative liquid media [7], making it challenging to achieve coordinated spatiotemporal operation at the nanoscale and convert it into visible, macroscopic, cell-like motion. Therefore, constructing artificial molecular machines that can work cooperatively to realize macroscopic non-equilibrium behaviors akin to living cells has become a key scientific challenge.

Recently, Deng and his colleagues [8] published a study titled “DNA photofluids show life-like motion” in *Nature Materials*, proposing an innovative solution to this problem. This study employs liquid–liquid phase separation technology [9] and artificial DNA molecular machines to construct, for the first time, a light-responsive fluidic material—DNA photofluids (Fig. 1). These photofluids can collect and amplify light-driven molecular motions, promoting the formation of non-equilibrium structures and enabling complex, cell-like behaviors at a macroscopic level. In this system, 2 light-driven dissipative processes—photoalignment and photofibrillation—play essential roles in coordinating molecular-scale motions into macroscopic deformation. Photoalignment guides the azobenzene moieties to orient along a specific direction, imparting directional deformation, while photofibrillation drives the assembly of DNA into nanofibers, enhancing structural stability and sustaining active motion.

**Fig. 1.**
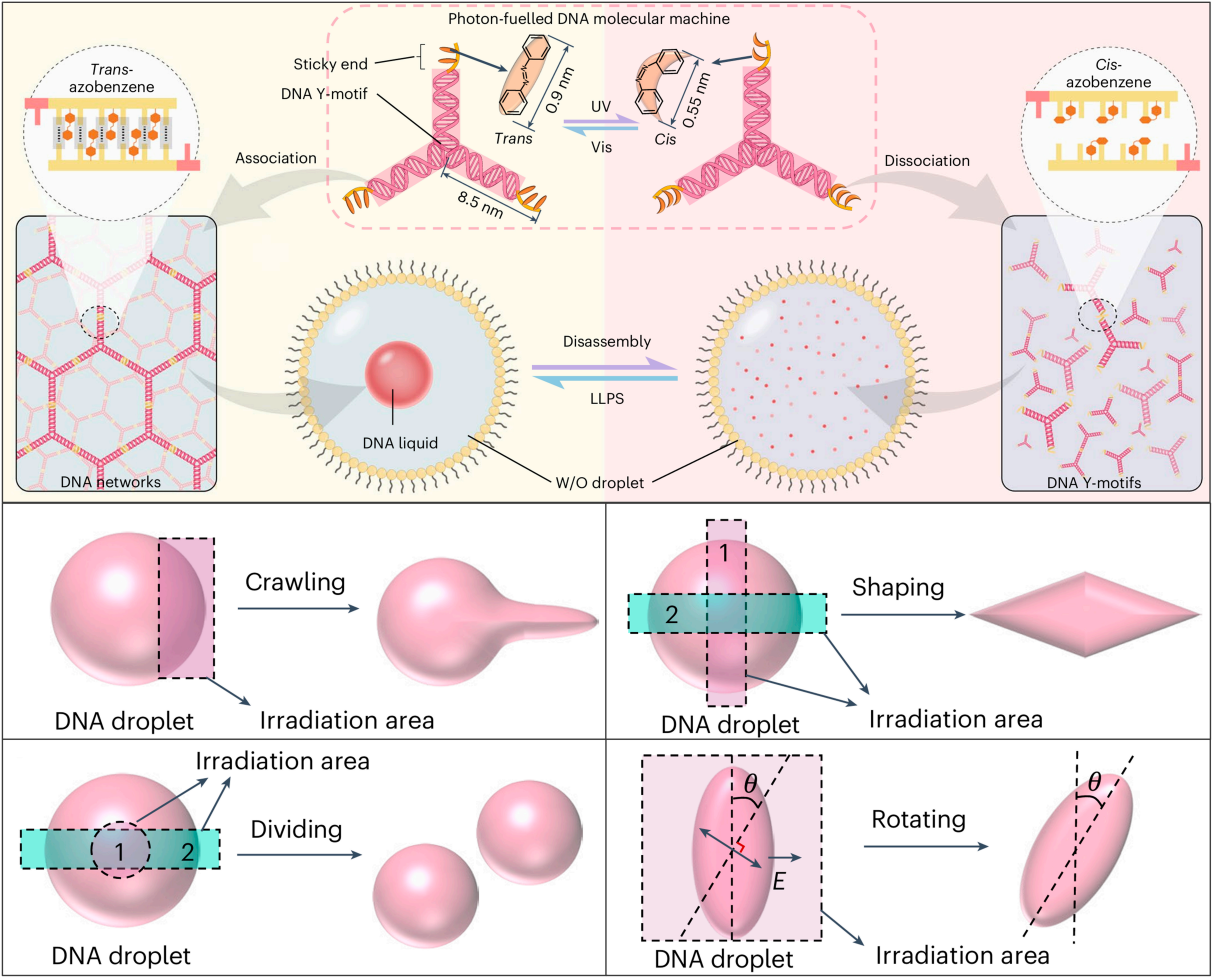
Design of Y-shaped DNA nanomachines with azobenzenes as nano-energy converters and photoinduced reversible assembly of DNA droplets. Copyright © 2025, Springer Nature [8].

DNA is a naturally occurring biomolecule whose strong programmability makes it an ideal material for constructing molecular machines [10,11]. Moreover, its ability to undergo base-pairing-driven self-assembly can facilitate liquid–liquid phase separation, a process in which a homogeneous solution separates into 2 or more liquid phases with distinct compositions and properties [12].

Among various DNA nanostructures, Y-shaped DNA strands—assembled from 3 single-stranded DNA oligonucleotides—possess sticky ends capable of driving DNA condensate formation through liquid–liquid phase separation. In this study, the authors introduced 2 to 3 azobenzene moieties at the sticky ends of the Y-shaped strands, serving as “energy conversion switches” and acting as light-responsive units [13]. Under ultraviolet (UV) light, the azobenzene groups switch from a rod-like trans conformation to a crescent-shaped cis conformation. In contrast, visible light triggers the reverse transformation, imparting the molecular machine with light-driven behavior [14,15]. When irradiated with UV light, the trans-to-cis conversion causes the dissociation of the sticky ends, macroscopically observed as the dissolution of DNA droplets in a water-in-oil system. Conversely, visible light induces the cis-to-trans transformation, restoring self-assembly and reinitiating liquid–liquid phase separation, with the reformation of submicrometer DNA droplets visible at the macroscopic level.

The authors found that irradiation with unpolarized visible light induces spherical DNA droplets to elongate into rod-like structures along the direction of light propagation; polarized light causes droplets to unfold perpendicularly into 2-dimensional liquid sheets; local illumination can generate pseudopod-like protrusions, enabling behaviors such as crawling, rotation, deformation, and localized division. The system can even function as a microactuator to create fluid vortices. All of these light-induced behaviors are highly reversible and programmable, and the deformation trajectories can be precisely directed through spatial light field distribution, showcasing the high flexibility and controllability of DNA photofluids in mimicking cellular life behaviors. Unlike conventional photoresponsive droplets and traditional molecular machines, which are often limited to simple behaviors and lack biologically relevant components, DNA photofluids establish an efficient coupling between energy conversion and macroscale ordered motion [16,17]. By enabling precise control and integrated simulation of multiple cell-like behaviors, they more closely mimic real cellular activities, representing a substantial advancement in the design of biomimetic materials for life-like behavior modeling.

Due to their ability to mimic cellular life behaviors, DNA photofluids serve as a vital foundation for constructing artificial cell models. Their innate ability to transduce energy into cell-like actions positions them as ideal candidates for investigating the transition from matter to life. In the future, integrating organelle-like functional modules into photofluids may enable the construction of more complete non-equilibrium artificial cells. Furthermore, this system holds great potential in various application fields. For instance, in drug delivery, external light fields could precisely control the division or deformation of DNA photofluid droplets to release therapeutic agents at specific times and locations, thereby improving targeting and treatment accuracy as part of an intelligent drug delivery system. Additionally, the ability of DNA photofluids to convert light into mechanical energy, combined with their spatial responsiveness and programmability, allows for behaviors such as pseudopodial motion, shape reconfiguration, and autonomous rotation. These properties make them promising candidates for developing light-controlled bioinspired soft robots. However, further research is required to assess the stability and functionality of DNA photofluid systems in complex biological environments.

In summary, this study innovatively integrates liquid–liquid phase separation with light-responsive molecular machines to construct an artificial molecular system—DNA photofluids—which can be precisely controlled by light in space and time and exhibit macroscopic behaviors mimicking those of living cells. This new material shows great potential in biomimetic innovative materials, microscale soft robotics, and artificial cell construction, offering a strong foundation and fresh perspectives for future research in artificial life systems and the development of intelligent dynamic materials.
